# Krüppel-like Factor-4-Mediated Macrophage Polarization and Phenotypic Transitions Drive Intestinal Fibrosis in THP-1 Monocyte Models In Vitro

**DOI:** 10.3390/medicina60050713

**Published:** 2024-04-26

**Authors:** Takuya Kanno, Takahito Katano, Takaya Shimura, Mamoru Tanaka, Hirotada Nishie, Shigeki Fukusada, Keiji Ozeki, Isamu Ogawa, Takahiro Iwao, Tamihide Matsunaga, Hiromi Kataoka

**Affiliations:** 1 Department of Gastroenterology and Metabolism, Nagoya City University Graduate School of Medical Sciences, 1 Kawasumi, Mizuho-cho, Mizuho-ku, Nagoya 467-8601, Japan; 2 Kajinoki Medical Clinic, 2340-1 Kawai, Kani, Gifu 509-0201, Japan; 3 Department of Clinical Pharmacy, Graduate School of Pharmaceutical Sciences, Nagoya City University, 3-1 Tanabe-dori, Mizuho-ku, Nagoya 467-8603, Japan; 4 Department of Molecular and Cellular Health Sciences, Graduate School of Pharmaceutical Sciences, Nagoya City University, 3-1 Tanabe-dori, Mizuho-ku, Nagoya 467-8603, Japan

**Keywords:** human-induced pluripotent stem cell, inflammatory bowel disease, interleukin-1, interleukin-10, interleukin-36, myofibroblast

## Abstract

*Background and Objectives*: Despite the fact that biologic drugs have transformed inflammatory bowel disease (IBD) treatment, addressing fibrosis-related strictures remains a research gap. This study explored the roles of cytokines, macrophages, and Krüppel-like factors (KLFs), specifically KLF4, in intestinal fibrosis, as well as the interplay of KLF4 with various gut components. *Materials and Methods*: This study examined macrophage subtypes, their KLF4 expression, and the effects of *KLF4* knockdown on macrophage polarization and cytokine expression using THP-1 monocyte models. Co-culture experiments with stromal myofibroblasts and a conditioned medium from macrophage subtype cultures were conducted to study the role of these cells in intestinal fibrosis. Human-induced pluripotent stem cell-derived small intestinal organoids were used to confirm inflammatory and fibrotic responses in the human small intestinal epithelium. *Results*: Each macrophage subtype exhibited distinct phenotypes and KLF4 expression. Knockdown of *KLF4* induced inflammatory cytokine expression in M0, M2a, and M2c cells. M2b exerted anti-fibrotic effects via interleukin (IL)-10. M0 and M2b cells showed a high migratory capacity toward activated stromal myofibroblasts. M0 cells interacting with activated stromal myofibroblasts transformed into inflammatory macrophages, thereby increasing pro-inflammatory cytokine expression. The expression of *IL-36α*, linked to fibrosis, was upregulated. *Conclusions*: This study elucidated the role of KLF4 in macrophage polarization and the intricate interactions between macrophages, stromal myofibroblasts, and cytokines in experimental in vitro models of intestinal fibrosis. The obtained results may suggest the mechanism of fibrosis formation in clinical IBD.

## 1. Introduction

Inflammatory bowel disease (IBD) is a chronic condition characterized by inflammation and ulceration of the gastrointestinal tract due to complex genetic, environmental, and gut microbiota interactions [1,2,3,4]. Crohn’s disease (CD) is a highly heterogeneous form of IBD characterized by distinct phenotypes such as perianal disease, fistulas, and strictures due to intestinal fibrosis. Although biologic drugs have revolutionized the management of CD, medical treatments targeting strictures have not been extensively researched, and there are currently no approved anti-stricture therapies [3,5,6,7].

Intestinal fibrosis is a reparative response to intestinal inflammation that involves tissue remodeling to preserve the integrity and function of the intestine [5]. Previous studies suggested that extracellular matrix (ECM) deposition plays a role in the development of intestinal fibrosis and strictures, with myofibroblasts playing a central role in ECM production [6,8]. Intestinal fibrosis can be triggered by various cytokines derived from immune cells located in the lamina propria [9]. Recently, interleukin (IL)-33 and IL-36, members of the IL-1 family, were reported as playing a role in intestinal fibrosis in studies using experimental mouse models [10,11]. However, the specific cytokines produced by immune cells that are responsible for promoting human intestinal fibrosis remain unclear. To elucidate the pathophysiology of IBD, it is essential to investigate the interactions between the intestinal epithelium, stromal myofibroblasts, and immune cells.

Macrophages are immune cells that play an important role in intestinal inflammation and fibrosis. Macrophages can be classified into subtypes of classically activated macrophages (M1) and alternatively activated macrophages (M2) depending on the microenvironment [12,13]. M1 cells, activated by interferon (IFN)-γ and lipopolysaccharide (LPS), exhibit microbicidal activity and promote robust Th1 responses mediated by IL-12. M2 cells can be subdivided into M2a cells (resulting from stimulation with IL-4 or IL-13), M2b cells (stimulated with immune complexes by IL-1β or LPS), and M2c cells (arising from IL-10, transforming growth factor (TGF)-β, or glucocorticoid exposure) [14]. M2-polarized macrophages critically support Th2-associated effector functions and play a role in the resolution of inflammation and fibrosis. However, it is unclear how the M2 subtypes interact with intestinal epithelial cells and stromal myofibroblasts to contribute to fibrosis during the process of intestinal mucosal fibrosis in IBD.

Krüppel-like factors (KLFs) are a subfamily of the zinc finger class of DNA-binding transcriptional regulators. KLF4 is normally expressed by differentiated epithelial cells of the intestine and contributes to intestinal epithelial homeostasis [15,16]. KLF4 is also a critical regulator of macrophage polarization in adipose tissue, exerting an inhibitory effect on bactericidal activity and accelerating the process of wound healing [17]. In the kidneys, KLF4 functions in mitigating injury and fibrosis by inhibiting the proinflammatory differentiation of renal-infiltrating macrophages [18]. However, the effect of alterations in cytokine expression via the regulation of M1/M2 subclass polarization modulated by KLF4 on fibrosis in the small intestine remains to be elucidated.

This study examined the potential mechanisms underlying the development of intestinal fibrosis in IBD. The results highlight the importance of crosstalk involving the intestinal epithelium and stromal myofibroblasts and immune cells modulated by KLF4 in the pathophysiology of IBD.

## 2. Materials and Methods

### 2.1. Cell Lines and Culture Methods

THP-1—a human leukemia monocytic cell line—cells (ATCC TIB202; ATCC) were cultured in a Roswell Park Memorial Institute (RPMI) 1640 medium supplemented with 10% fetal bovine serum (FBS) and 1% penicillin–streptomycin–amphotericin B suspension (Wako Pure Chemical Industries, Osaka, Japan) at 37 °C under 5% CO_2_.

THP-1 cells were treated with 320 nmol/mL phorbol myristate acetate for 6 h to differentiate into M0 macrophages. The resulting M0 macrophages were stimulated with 20 ng/mL IFN-γ (Wako Pure Chemical Industries, Osaka, Japan) and 100 ng/mL LPS (Sigma Aldrich, St. Louis, MI, USA) for 18 h to induce differentiation into M1 macrophages or stimulated with 20 ng/mL IL-4 (Wako Pure Chemical Industries, Osaka, Japan) and 20 ng/mL IL-13 (Wako Pure Chemical Industries, Osaka, Japan) for 18 h to induce differentiation into M2a macrophages. To obtain M2b macrophages, LPS (100 ng/mL) was added for 18 h to cells cultured in wells of plates coated with γ-globulin (50 μg/mL) (Sigma Aldrich, St. Louis, MI, USA). M0 macrophages were stimulated with 0.5 ng/mL TGF-β (Wako Pure Chemical Industries, Osaka, Japan) for 18 h to induce differentiation into M2c macrophages.

Normal human intestinal myofibroblast (IMF) cells (CC-2902; Lonza, Basel, Switzerland) were cultured in a smooth muscle basal medium supplemented with 5% FBS and growth factors (insulin, human epidermal growth factor (EGF)–β, and human fibroblastic growth factor (FGF)) and gentamicin and amphotericin B (CC-4149, Lonza) at 37 °C under 5% CO_2_. IMFs were activated by culturing with 5 ng/mL TGF-β in an RPMI 1640 medium for 24 h.

### 2.2. Human Induced Pluripotent Stem (iPS) Cell-Derived Small Intestinal Organoids (HIOs)

A human iPS cell line (Windy) was used in this study. Human iPS cells were cultured as described previously and induced to differentiate from iPS cells to HIOs based on previously reported methods, with some modifications [19,20]. Briefly, iPS cells were exposed to Activin A for 3 days to generate endoderm-like cells, which were then induced to differentiate into hindgut-like cells by exposure to FGF4 and CHIR99021 for 4 days. Hindgut-like cells were cultured to 90% confluence on laminin-511 E8 fragment-coated plates for 72–96 h in a medium containing 5% FBS and 100 ng/mL EGF, 3 μM CHIR99021, 500 nM A-83-01, 10 μM Y-27632, and 30 ng/mL FGF2. Next, cells (5.0 × 10^6^) were seeded on an EZSPHERE plate (AGC Techno Glass Co., Ltd., Shizuoka, Japan) and cultured for 3 days to generate spheres. After preparation, spheres were cultured in a medium containing 3% Matrigel for 6–18 days to generate HIOs.

### 2.3. Scratch-Wound Healing Assay

Induced polarized macrophages were washed with PBS and cultured with RPMI1640. After 24 h, the cell-free supernatants were collected and used for IMF stimulation in a scratch-wound healing assay. IMFs were transferred into 12-well plates and cultured in an incubator at 37 °C under 5% CO_2_. When the cells reached confluence, a straight wound was made in the cell layer by scratching with a sterile 200 μL pipette tip. Then, 1 mL of RPMI with 1% FBS (blank control group) or supernatant from each macrophage subtype with 1% FBS was added to each well. The cells were observed at 0 h and 24 h, and then the scratched area was measured using ImageJ software 1.54 (National Institutes of Health, Bethesda, MD, USA), and the scratched area healing rate was calculated, defined as (scratched area at 0 h—scratched area at 24 h)/(scratched area at 0 h).

### 2.4. Transwell Migration Assay

IMF cells and TGF-β-activated IMF cells were transferred into 24-well plates and cultured at 37 °C under 5% CO_2_. When the cells reached 80% confluence, a Transwell cell culture insert (4-µm pore size, Sartorius) was transferred into the plate, and 0.5 mL of macrophage suspension (1 × 10^5^ cells/mL) was placed into the cell culture insert and incubated for 24 h. The medium in the upper and lower chambers of the cell culture insert was removed. Macrophages on the surface of the upper membrane were wiped off using cotton swabs. After washing with phosphate-buffered saline (PBS), migratory macrophages were fixed with 4% paraformaldehyde for 30 min. The cells were then stained with 0.1% crystal violet for 30 min. The macrophages were washed with PBS and air-dried. The migratory macrophages were counted in 3 low-power fields of microscopy images.

### 2.5. RNA Extraction, Reverse Transcription, and Real-Time PCR

The mRNA expression levels of the *CD163*, *MRC1*, *TNF-α*, *IL-1β*, *IL-6*, *IL-10*, *TGF-β*, *KLF4*, *IL-36α*, and *GAPDH* genes in macrophages, *αSMA* in IMFs, and *αSMA* and *IL-36γ* in HIOs were measured using real-time reverse transcription PCR (RT-PCR). *GAPDH* was used as an endogenous control for data standardization. mRNA was reverse transcribed into single-stranded cDNA using a High-Capacity cDNA Reverse Transcription kit (Applied Biosystems, Waltham, MI, USA). TaqMan gene expression assays for *CD163* (Hs.PT.58.3564170), MRC1 (Hs.PT.58.15093573), *TNF-α* (Hs1113624_g1), *IL-1β* (Hs.PT.58.1518186), *IL-6* (Hs00985639_m1), *IL-10* (Hs.PT,58.2807216), *TGF-β* (Hs.PT.58.3981395), *KLF4* (Hs00358836_m1), *IL-36α* (Hs.PT.58.22339976), *αSMA* (Hs.PT.56a.2542642), and *GAPDH* (Hs99999905_m1) were purchased from Integrated DNA Technologies (IDT, Coralville, IA, USA) and Applied Biosystems. Quantitative RT-PCR analyses were performed using an ABI Fast Real-Time PCR system (Applied Biosystems). The mRNA expression levels were normalized to those of the housekeeping gene encoding glyceraldehyde-3-phosphate dehydrogenase (GAPDH). Assays were performed in triplicate, and results from at least three independent experiments are presented. Relative mRNA levels were calculated using the ΔΔCt method.

### 2.6. Western Blot Analysis

Cells were dissolved in lysis buffer, and 10 µL of each protein sample was fractionated on polyacrylamide gels (TGX™ FastCast™ Acrylamide Kit; Bio-Rad Laboratories, Hercules, CA, USA) and then electroblotted onto nitrocellulose membranes. The membranes were blocked and incubated with primary antibodies against β-actin (FUJIFILM Wako Pure Chemical Corp., Osaka, Japan) or KLF4 (Novus Biologicals, Littleton, CO, USA), and then the membranes were incubated with horseradish peroxidase-conjugated secondary antibody. The membranes were then treated with enhanced chemiluminescence detection reagents (Amersham™; Cytiva, Marlborough, MA, USA), and chemiluminescent signals were visualized as bands using a LAS 4000 mini analyzer (Cytiva).

### 2.7. Transfection

Small interfering RNA (siRNA) transfection was carried out using Lipofectamine RNAi-MAX (Thermo Fisher Scientific, Waltham, MA, USA). M0, M2a, and M2c macrophages were transfected with siRNA using a siGENOME non-targeting siRNA (NT) control pool and a human KLF4 siRNA SMART pool (Dharmacon, Lafayette, CO, USA). Forty-eight hours after transfection, macrophages were cultured with the control medium for 24 h. The assessment for cells transfected with the delivery reagent alone (mock) and NT (negative control) conditions showed no significant changes in the expression levels of *KLF4*. This provided a valuable control to ensure that any observed effects were not attributed to the delivery reagent alone.

### 2.8. Immunofluorescence Staining

Immunofluorescence staining was performed using antibodies against the following antigens: αSMA (1:500; ab32575, abcam, Cambridge, UK) and KLF4 (1:100; JF98-08, Novus Biologicals). Macrophages and IMFs grown on a glass plate were fixed with 4% paraformaldehyde and then incubated with PBS containing 0.2% Triton X-100 and blocked with a blocking reagent (Nichirei Bio Science, Tokyo, Japan). The samples were then reacted with primary antibodies at room temperature for 1 h, washed with PBS, and then incubated with secondary antibodies at room temperature for 1 h. Nuclei were counterstained using 4′,6-diamidino-2-phenylindole (DAPI). Furthermore, macrophages were attached to glass slides. Confocal images were captured using a FV1000 laser scanning microscope (Olympus Life Science, Tokyo, Japan).

### 2.9. Statistical Analysis

Statistical analysis was carried out using GraphPad Prism software, version 9.2.0 for Windows (GraphPad Software, San Diego, CA, USA). The two-tailed Student’s *t*-test, two-tailed Dunnett’s test, and Tukey’s test were used, as appropriate. The number of samples is indicated by “n”. At least two independent replicates were conducted for all experiments. Data are shown as the mean ± standard deviation (SD). A *p* value < 0.05 was considered statistically significant.

## 3. Results

### 3.1. Phenotypes of Macrophage Subtypes and Differences in KLF4 Expression

**Figure 1:**
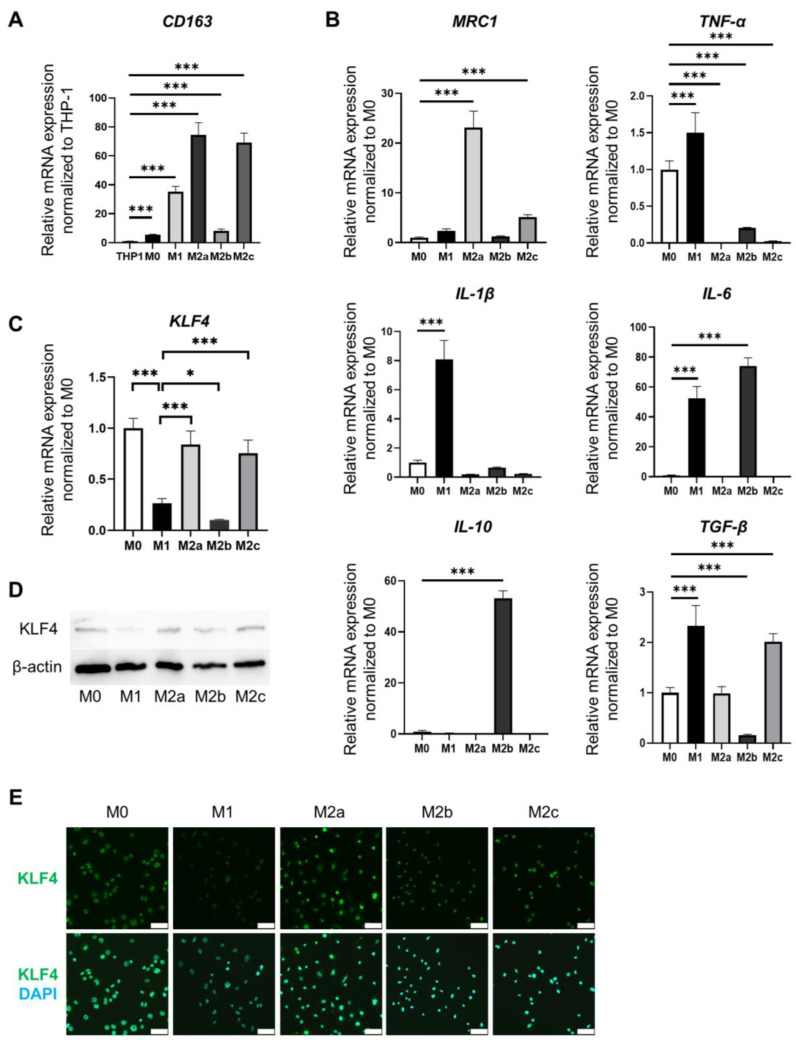
Phenotypes of each subtype of macrophages and differences in KLF4 expression. The mRNA expression levels were normalized to those of GAPDH. (**A**). Relative mRNA expression of *CD163* in THP-1 cells and each macrophage subtype. The mRNA levels were expressed relative to the mean value of THP1; mean ± SD; *n* = 6. Statistical analysis was performed using two-tailed Dunnett’s test; *** *p* < 0.001. (**B**). Relative mRNA expression of *MRC1*, *TNF-α*, *IL-1β*, *IL-6*, *IL-10*, and *TGF-β* in each macrophage subtype. The mRNA levels were expressed relative to the mean value of M0; mean ± SD; *n* = 6. Statistical analysis was performed using two-tailed Dunnett’s test; *** *p* < 0.001. (**C**). Relative mRNA expression of *KLF4* in each macrophage subtype. The mRNA levels were expressed relative to the mean value of M0; mean ± SD; *n* = 6. Statistical analysis was performed using two-tailed Dunnett’s test; * *p* < 0.05, *** *p* < 0.001. (**D**). Protein expression of KLF4 in each macrophage subtype assessed by Western blotting. (**E**). Immunofluorescence staining for KLF4 (green) in each macrophage subtype. Nuclei were counterstained with DAPI (blue). Scale bars: 50 µm. DAPI, 4′,6-diamidino-2-phenylindole.

Differentiated and adherent cells were designated M0, M1, M2a, M2b, or M2c by applying the previously reported stimulation to THP-1 cells. The expression of CD163 as a macrophage marker was significantly elevated in M0, M1, M2a, M2b, and M2c cells compared to its expression in THP-1 cells (Figure 1A). The phenotypes of differentiated macrophages were determined based on gene expression profiles for each macrophage subtype (Figure 1B). *MRC1* expression was significantly elevated in M2a and M2c cells compared to its expression in M0 cells (*p* < 0.001 and *p* < 0.001, respectively). *TNF-α* expression was significantly elevated in M1 cells compared to its expression in M0 cells (*p* < 0.001). *IL-1β* expression was significantly elevated in M1 cells compared to its expression in M0 cells (*p* < 0.001). *IL-6* expression was significantly elevated in M1 and M2b cells compared to its expression in M0 cells (*p* < 0.001 and *p* < 0.001, respectively). *IL-10* expression was significantly elevated in M2b cells compared to its expression in M0 cells (*p* < 0.001). *TGF-β* expression was significantly elevated in M1 and M2c cells compared to its expression in M0 cells (*p* < 0.001 and *p* < 0.001, respectively). These analyses of characteristic markers in M1 cells in our study identified TNF-α and IL-1β in M1 cells, MRC1 in M2a cells, IL-10 in M2b cells, and TGF-β and MRC1 in M2c cells as markers of the characteristic phenotype of each differentiated macrophage subtype (Figure 1B).

Next, we examined macrophage-subtype-related differences in the expression of KLF4, which is reportedly involved in the induction of macrophage polarity (Figure 1C–E). *KLF4* expression in M1 cells was significantly suppressed compared to M0 cells (*p* < 0.001) (Figure 1C). *KLF4* expression in M2a and M2c cells was significantly higher than in M1 cells (*p* < 0.001 and *p* < 0.001, respectively) (Figure 1C). By contrast, *KLF4* expression in M2b cells was significantly lower than that in M1 cells (*p* < 0.05). Analysis of KLF4 protein expression by Western blotting showed similar results to mRNA expression (Figure 1D). KLF4 protein expression was examined in each macrophage subtype using immunofluorescence staining. The fluorescence intensity results were also consistent with the results of mRNA expression and Western blotting analyses (Figure 1E). Hence, these results revealed that each macrophage subtype that differentiated from THP-1 cells expressed different characteristic phenotypic markers and expressed KLF4 at different levels.

### 3.2. Effect of KLF4 Knockdown on Macrophage Polarization and Inflammatory Phenotype Induction

**Figure 2:**
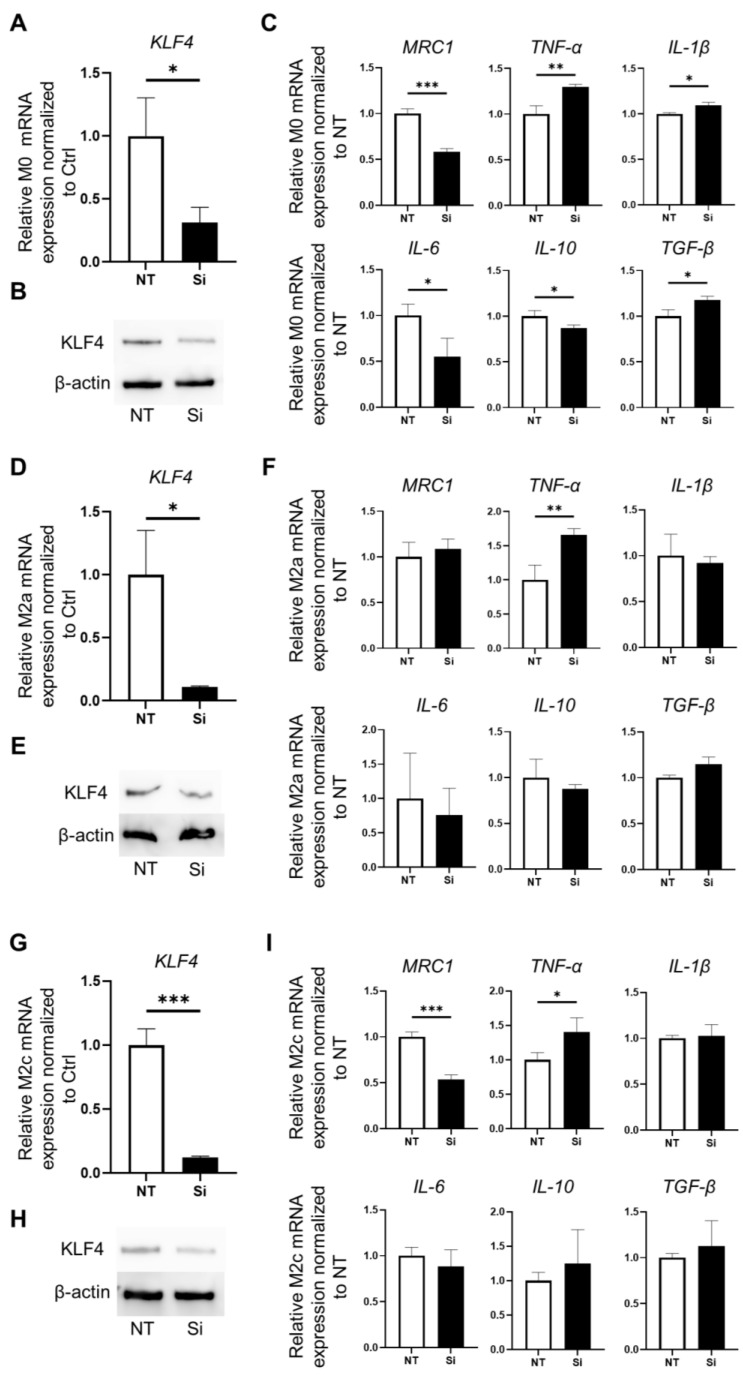
Phenotypic changes resulting from *KLF4* knockdown in polarized macrophages: M0 cells (**A**–**C**), M2a cells (**D**–**F**), and M2c cells (**G**–**I**). (**A**). Relative expression of *KLF4* mRNA in M0 cells. *KLF4* was effectively silenced using siRNA. Relative expression of *KLF4* mRNA was significantly decreased in the Si compared with the NT (*p* < 0.05); mean ± SD; *n* = 3. Statistical analysis was performed using two-tailed Student’s *t*-test; * *p* < 0.05. (**B**). Relative expression of KLF4 protein in *KLF4*-knockdown M0 cells assessed by Western blotting. (**C**). Relative expression of *MRC1*, *TNF-α*, *IL-1β*, *IL-6*, *IL-10*, and *TGF-β* mRNA in *KLF4*-knockdown M0 cells; mean ± SD; *n* = 3. Statistical analysis was performed using two-tailed Student’s *t*-test; * *p* < 0.05, ** *p* < 0.01, *** *p* < 0.001. (**D**). Relative expression of *KLF4* mRNA. *KLF4* expression in M2a cells was effectively silenced using siRNA. *KLF4* expression was significantly decreased in the Si compared with the NT (*p* < 0.05); mean ± SD; *n* = 3. Statistical analysis was performed using two-tailed Student’s *t*-test; * *p* < 0.05. (**E**). Relative expression of KLF4 protein in *KLF4*-knockdown M2a cells assessed by Western blotting. (**F**). Relative expression of *MRC1*, *TNF-α*, *IL-1β*, *IL-6*, *IL-10*, and *TGF-β* mRNA in *KLF4*-knockdown M2a (Si M2a) cells. Relative expression of *TNF-α* mRNA was significantly elevated in the Si M2a compared with the NT M2a cells (*p* < 0.01); mean ± SD; *n* = 3. Statistical analysis was performed using two-tailed Student’s *t*-test; ** *p* < 0.01. (**G**). Relative expression of *KLF4* mRNA in M2c cells was effectively silenced using siRNA. Relative expression of *KLF4* mRNA was significantly decreased in Si M2c cells compared with NT M2c cells (*p* < 0.001); mean ± SD; *n* = 3. Statistical analysis was performed using two-tailed Student’s *t*-test; *** *p* < 0.001. (**H**). Relative expression of KLF4 protein in *KLF4*-knockdown M2c cells assessed by Western blotting. (**I**). Relative expression of *MRC1*, *TNF-α*, *IL-1β*, *IL-6*, *IL-10*, and *TGF-β* mRNA in *KLF4*-knockdown M2c cells; mean ± SD; *n* = 3. Statistical analysis was performed using two-tailed Student’s *t*-test; * *p* < 0.05, *** *p* < 0.001. Ctrl: control, NT: macrophages silenced by non-targeting control siRNA, Si: macrophages silenced by KLF4-siRNA.

Knockdown of *KLF4* in M0 macrophages was achieved using siRNA to confirm the role of KLF4 in macrophage polarization. In *KLF4*-knockdown M0 (Si M0) cells, *KLF4* expression was significantly lower than in non-targeted siRNA-transfected M0 (NT M0) cells and normal M0 cells (*p* < 0.05) (Figure 2A). *KLF4*-knockdown M0 cells showed lower protein expression of KLF4 by Western blot analysis (Figure 2B). *TNF-α*, *IL-1β*, and *TGF-β* expression in Si M0 cells was significantly higher than in NT M0 cells (*p* < 0.01, *p* < 0.05, and *p* < 0.05, respectively). By contrast, *MRC1*, *IL-6*, and *IL-10* expression in Si M0 cells was significantly downregulated compared to NT M0 cells (*p* < 0.001, *p* < 0.05, and *p* < 0.05, respectively) (Figure 2C).

To confirm the correlation between KLF4 and phenotypic markers characteristic of polarized differentiated macrophages, we performed knockdown of *KLF4* in M2a and M2c macrophages, which express higher levels of *KLF4*. In *KLF4*-knockdown M2a (Si M2a) cells, *KLF4* expression was significantly lower than in non-targeted siRNA-transfected M2a (NT M2a) cells (*p* < 0.05) (Figure 2D). Western blot analysis showed lower protein expression of KLF4 in Si M2a cells (Figure 2E). *TNF-α* expression in Si M2a cells was significantly elevated compared to NT M2a cells (*p* < 0.01) (Figure 2F). *KLF4*-knockdown M2c (Si M2c) cells showed significantly lower mRNA and protein expression of KLF4 compared to non-target siRNA-transfected M2c (NT M2c) cells (*p* < 0.001) (Figure 2G,H). *TNF-α* expression in Si M2c cells was significantly elevated compared to NT M2c cells (*p* < 0.05). By contrast, *MRC1* expression in Si M2c cells was significantly downregulated compared to NT M2c cells (*p* < 0.001) (Figure 2I). *KLF4* knockdown induced *TNF-α* expression in all M0, M2a, and M2c cells that expressed *KLF4*. With M0 cells in particular, *KLF4* knockdown also downregulated the expression of *MRC1*, a marker characteristic of M2a cells, and upregulated the expression of *IL-1β*, which is highly expressed by M1 cells. These results suggest that KLF4 plays a role in regulating macrophage polarity, particularly with regard to the inflammatory macrophage phenotype.

### 3.3. Macrophage Subtypes Modulate Intestinal Fibrosis

**Figure 3:**
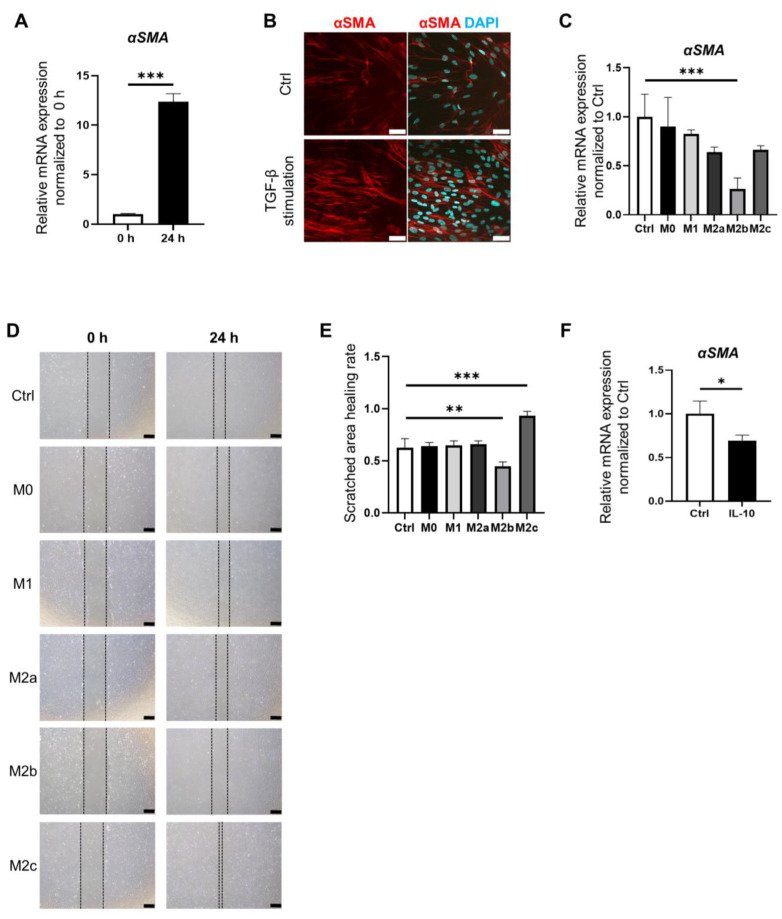
Macrophage subtypes modulate intestinal fibrosis. (**A**). IMFs were activated by stimulation with TGF-β for 24 h. Relative expression of *αSMA* mRNA in IMFs at 0 h and 24 h. Relative expression of *αSMA* mRNA in IMFs was significantly increased at 24 h compared with 0 h (*p* < 0.001); mean ± SD; *n* = 3. Statistical analysis was performed using two-tailed Student’s *t*-test; *** *p* < 0.001. (**B**). Immunofluorescence staining for αSMA (red) in control IMFs and IMFs stimulated with TGF-β for 24 h. Nuclei were counterstained with DAPI (blue). Scale bars: 50 µm. (**C**). IMFs were cultured with a conditioned medium prepared from the culture supernatant of each macrophage subtype. The expression of *αSMA* was significantly decreased in IMFs cultured with M2b medium compared with the control (blank; RPMI with 1% FBS) (*p* < 0.001); mean ± SD; *n* = 3. Statistical analysis was performed using two-tailed Dunnett’s test; *** *p* < 0.001. (**D**). Macroscopic images at 0 h and 24 h of scratch-wound healing assay results. IMFs were cultured with conditioned medium prepared from culture supernatant of each macrophage subtype. Scale bars: 500 µm. (**E**). Scratched area healing rate of scratch-wound healing assay. Scratched wound area was measured at 0 h and 24 h. Scratched area healing rate was calculated as (scratched area at 0 h—scratched area at 24 h)/(scratched area at 0 h) using ImageJ software 1.54. The scratched area healing rate was significantly decreased in IMFs cultured with the M2b medium compared with the control (*p* < 0.05). The scratched area healing rate was significantly increased in IMFs cultured with M2c medium compared with the control (*p* < 0.01); mean ± SD; *n* = 3. Statistical analysis was performed using two-tailed Dunnett’s test; ** *p* < 0.01, *** *p* < 0.001. (**F**). Relative expression of *αSMA* mRNA in IMFs supplemented with IL-10. The expression of *αSMA* was significantly decreased in IMFs supplemented with IL-10 compared with the control (*p* < 0.05). mean ± SD; *n* = 3. Statistical analysis was performed using two-tailed Student’s *t*-test; * *p* < 0.05. Ctrl: control.

In order to elucidate the roles of individual macrophage subtypes in the development of fibrosis in the intestinal epithelium, we conducted co-culture experiments involving intestinal stromal myofibroblasts (IMFs) and conditioned a medium prepared from the culture supernatant of each macrophage subtype. The expression of *αSMA* in IMFs was markedly elevated at 24 h after the addition of TGF-β (Figure 3A). Immunofluorescence staining for αSMA showed higher fluorescence intensity in IMFs activated by TGF-β (Figure 3B). To examine the association between macrophage subtypes and activation of IMFs, culture supernatants of each macrophage subtype were collected and used for the co-culture of IMFs for 24 h. The expression of *αSMA* in IMFs co-cultured with M2b supernatant was significantly suppressed compared to the control (*p* < 0.001) (Figure 3C).

A scratch-wound healing assay was performed to identify the interactions among different macrophage subtypes that stimulate the proliferation of IMFs. IMFs were co-cultured with the supernatant of each macrophage subtype. The scratched area healing rate of M2b cells was significantly lower than that of control cells (*p* < 0.05). By contrast, the scratched area healing rate of M2c cells was significantly higher than that of control cells (*p* < 0.01) (Figure 3D,E) (Supplementary Table S1). These findings suggest that M2b exerts inhibitory effects on IMF activation, whereas M2c cells appear to stimulate IMF activation.

To identify the factor(s) that inhibit IMF activation, we focused on IL-10, which is highly expressed by M2b cells. The expression of *αSMA* in IMFs stimulated with IL-10 was significantly suppressed compared to control cells (*p* < 0.05) (Figure 3F). Hence, M2b cells might suppress the development of small intestinal fibrosis via IL-10.

### 3.4. Macrophage Polarization and Fibrosis Modulation in the Intestinal Stromal Myofibroblast Environment

**Figure 4:**
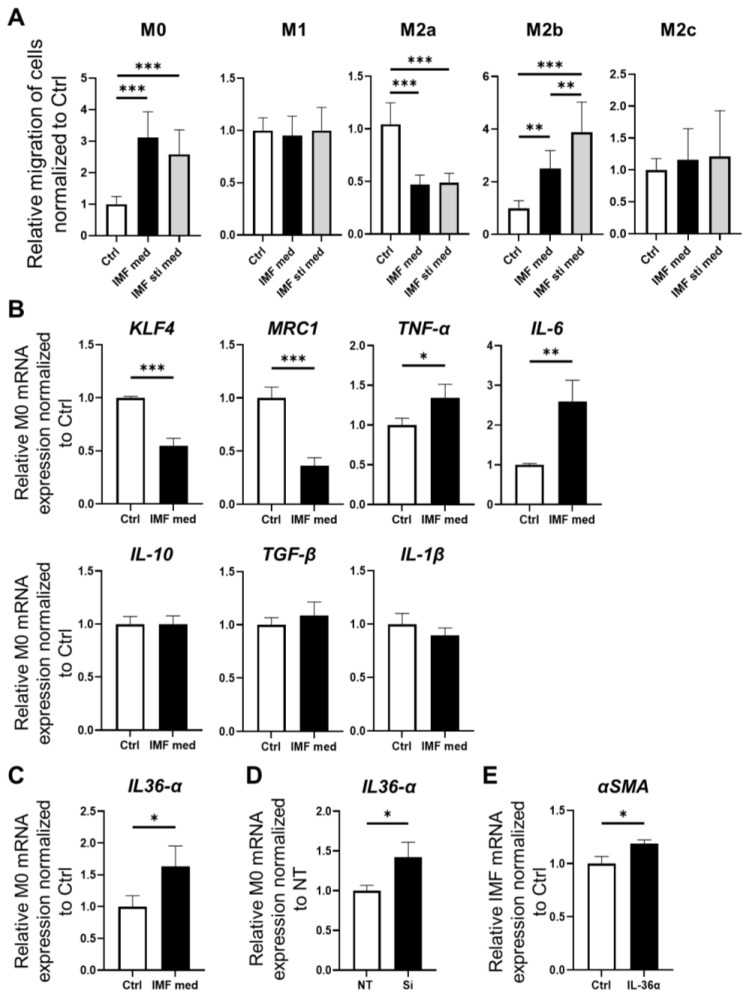
Interactions between macrophages and fibrosis in the presence of IMFs. (**A**). Migratory capacity of macrophages was analyzed using a Transwell migration assay. Macrophages were co-cultured with IMFs (IMF-med) or activated IMFs stimulated with TGF-β (IM-sti-med). The number of migrated M0 cells was significantly increased in IMF-med and IMF-sti-med compared with the blank control (IMF-med and IMF-sti-med vs. Ctrl, *p* < 0.001). The number of migrated M2a cells was significantly decreased in both IMF-med and IMF-sti-med compared with the blank control (*p* < 0.001). The number of migrated M2b cells was significantly increased in IMF-med and IMF-sti-med compared with the blank control (*p* < 0.001); mean ± SD; *n* = 9. Statistical analysis was performed using Tukey’s test; ** *p* < 0.01, *** *p* < 0.001. (**B**). Relative expression of *KLF4*, *MRC1*, *TNF-α*, *IL-6*, *IL-10*, *TGF-β*, and *IL-1β* mRNA in M0 cells cultured with the IMF supernatant. Relative expression of *KLF4* mRNA was significantly decreased in M0 cells cultured with the IMF supernatant compared with the blank control (*p* < 0.001). Relative expression of *MRC1* mRNA was significantly decreased in M0 cells cultured with the IMF supernatant compared with the control (*p* < 0.001). Relative expression of *TNF-α* mRNA was significantly increased in M0 cells cultured with the IMF supernatant compared with the control (*p* < 0.05). Relative expression of *IL-6* mRNA was significantly increased in M0 cells cultured with the IMF supernatant compared with the control (*p* < 0.01); mean ± SD; *n* = 3. Statistical analysis was performed using two-tailed Student’s *t*-test; * *p* < 0.05, ** *p* < 0.01, *** *p* < 0.001. (**C**). Relative expression of *IL-36α* mRNA in M0 cells cultured with the IMF supernatant. The expression of *IL-36α* mRNA was significantly increased in M0 cells cultured with the IMF supernatant compared with the blank control (*p* < 0.05); mean ± SD; *n* = 3. Statistical analysis was performed using two-tailed Student’s *t*-test; * *p* < 0.05. (**D**). Relative expression of *IL-36α* mRNA in normal M0 (control) and *KLF4*-knockdown M0 cells. The expression of *IL-36α* increased significantly in the Si compared with the NT (*p* < 0.05); mean ± SD; *n* = 3. Statistical analysis was performed using two-tailed Student’s *t*-test; * *p* < 0.05. (**E**). Relative expression of *αSMA* mRNA in normal IMFs (control) and IMFs supplemented with IL-36α. The expression of *αSMA* was significantly increased in IMFs supplemented with IL-36α compared with the control (*p* < 0.05); mean ± SD; *n* = 3. Statistical analysis was performed using two-tailed Student’s *t*-test; * *p* < 0.05. Ctrl: control, NT: macrophage was silenced by non-targeting control siRNA, Si: macrophage was silenced by KLF4-siRNA macrophage.

To investigate the migratory capacity of polarized macrophages in the intestinal stromal myofibroblast environment, we conducted a Transwell migration assay (Figure 4A). The number of infiltrating M0 macrophages was significantly increased in both the presence of IMFs and activated IMFs compared to the control (IMF-med and IMF-sti-med vs. Ctrl, *p* < 0.001). The number of infiltrating M2a macrophages was significantly decreased in both the presence of IMFs and activated IMFs compared to the control (IMF-med and IMF-sti-med vs. Ctrl, *p* < 0.001). The number of infiltrating M2b macrophages was significantly increased in both the presence of IMFs and activated IMFs compared to the control (IMF-med vs. Ctrl, IMF-sti-med vs. Ctrl, and IMF-sti-med vs. IMF-med; *p* < 0.01, *p* < 0.001, and *p* < 0.001, respectively). The number of infiltrating M1 and M2c cells did not change in the presence of IMFs (Figure 4A). These results suggest that among the macrophage subtypes, M0 and M2b accumulate in the activated intestinal stromal myofibroblast environment, playing an important role in intestinal fibrosis.

We previously demonstrated the fibrosis-inhibiting role of IL-10, which is highly expressed by M2b cells. We therefore co-cultured M0 cells with conditioned medium of IMFs to elucidate the role of accumulated M0 cells in fibrosis within the stromal intestinal myofibroblast environment. Remarkably, *KLF4* expression by M0 cells cultured with the IMF supernatant (IMF-sup-M0) was significantly suppressed compared to control cells (*p* < 0.001). In addition, *MRC1* expression in IMF-sup-M0 cells was significantly lower than in control cells (*p* < 0.001). *TNF-α* and *IL-6* expression in IMF-sup-M0 cells was significantly elevated compared to control cells (*p* < 0.05 and *p* < 0.01, respectively). These results thus suggest that M0 cells in the presence of IMFs are transformed into inflammatory macrophages (Figure 4B). Inflammatory macrophages have been implicated as playing a role in the fibrotic response of the intestinal epithelium, and IL-36, a cytokine of the IL-1 family, is reportedly involved in intestinal fibrosis. Therefore, we hypothesized that macrophages polarized from M0 to an inflammatory phenotype in the intestinal stromal myofibroblast environment might induce the expression of IL-1 family members along with inflammatory cytokines. The expression of *IL-36α* in IMF-sup-M0 cells was significantly higher than in control cells (*p* < 0.05) (Figure 4C). To investigate the correlation between KLF4 and IL-36α, we examined the changes in *IL-36α* expression following *KLF4* knockdown in M0 cells. *IL-36α* expression in *KLF4*-knockdown M0 cells was significantly elevated compared to NT M0 cells (*p* < 0.05) (Figure 4D). When IMFs were treated with IL-36α, the expression of *αSMA* was significantly increased (*p* < 0.05) (Figure 4E). These findings suggest that undifferentiated M0 cells in the intestinal stromal myofibroblast environment shift toward an inflammatory macrophage phenotype, expressing inflammatory cytokines such as TNF-α and IL-6, with suppressed KLF4 expression. Furthermore, these data suggest that inflammatory macrophages derived from M0 cells in the intestinal stromal myofibroblast environment play a role in promoting fibrosis through the upregulation of IL-36α expression.

### 3.5. Characterization of Inflammatory and Fibrotic Responses in an IBD Model Using HIOs

**Figure 5:**
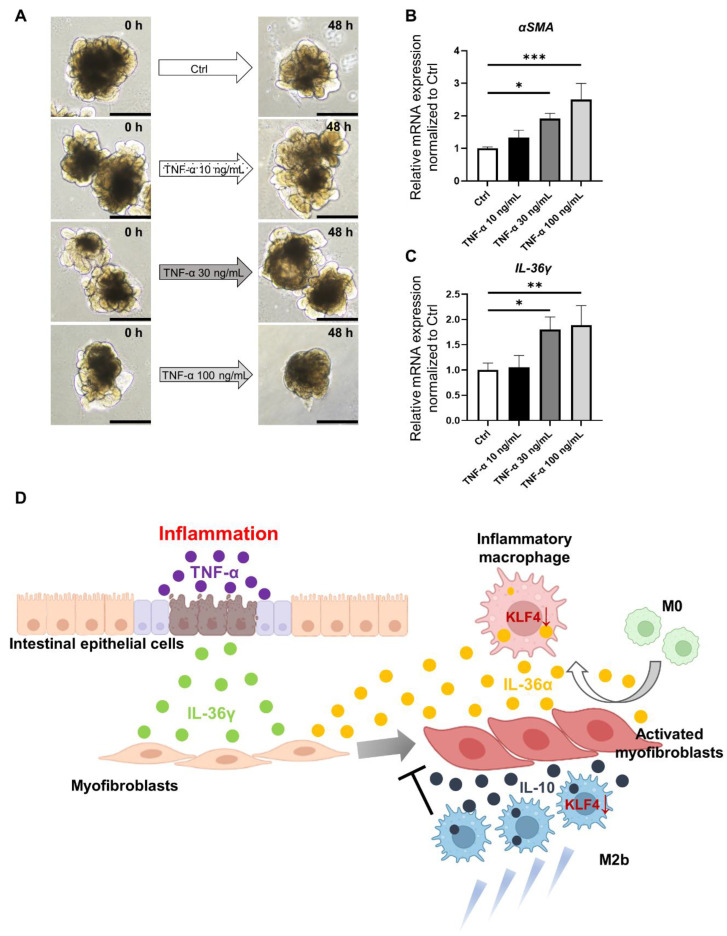
Analysis in an ex vivo model reconstituting IBD using HIOs. (**A**). Macroscopic images of organoids stimulated with TNF-α. Scale bars: 500 µm. (**B**). Relative expression of *αSMA* mRNA in control organoids (without TNF-α addition) and organoids supplemented with TNF-α at 30 ng/mL and 100 ng/mL. Relative expression of *αSMA* mRNA was significantly increased in the TNF-α 30 ng/mL and TNF-α 100 ng/mL groups compared with the control (*p* < 0.05 and *p* < 0.001, respectively); mean ± SD; *n* = 3. Statistical analysis was performed using two-tailed Dunnett’s test; * *p* < 0.05, *** *p* < 0.001. (**C**). Relative expression of *IL-36γ* mRNA in control organoid and organoids supplemented with TNF-α at each concentration. Relative expression of *IL-36γ* mRNA was significantly increased in the 30 ng/mL and 100 ng/mL groups compared with the control (*p* < 0.05 and *p* < 0.01, respectively); mean ± SD; *n* = 3. Statistical analysis was performed using two-tailed Dunnett’s test; * *p* < 0.05, ** *p* < 0.01. (**D**). Interactions between the small intestinal epithelium, intestinal stromal myofibroblasts, and macrophages and cytokines involved in intestinal inflammation and fibrosis. Ctrl: control, HIOs: human-induced pluripotent stem cell-derived small intestinal organoids, IBD: inflammatory bowel disease.

Finally, we established a model mimicking IBD using HIOs. We confirmed the inflammatory and fibrotic responses in the human small intestinal epithelium using this model. The HIOs demonstrated epithelial damage and disruption of the 3D structure in a TNF-α concentration-dependent manner (Figure 5A). Stimulation with TNF-α at 30 ng/mL and 100 ng/mL significantly upregulated *αSMA* expression in HIOs (*p* < 0.05 and *p* < 0.001, respectively) (Figure 5B). To investigate the involvement of the IL-1 family in the inflamed epithelium, we initially examined *IL-36α* expression in post-inflammatory HIOs stimulated with TNF-α, but no *IL-36α* expression in the HIO epithelium was observed. However, when we examined *IL-36γ*, another member of the IL-1 family, expression of *IL-36γ* was significantly elevated in inflamed epithelial HIOs stimulated with TNF-α at 30 ng/mL and 100 ng/mL (*p* < 0.05 and *p* < 0.01, respectively) (Figure 5C).

Figure 5D illustrates the network of the small intestinal epithelium, stromal myofibroblasts, and macrophages in IBD, as suggested by the results obtained in this study. Inflammatory activation of the small intestinal epithelium, along with epithelial damage, leads to the activation of stromal myofibroblasts, and the resulting release of IL-36γ from the epithelium is also implicated in fibrosis. Among polarized differentiated macrophages, the data suggest that M2b cells exert inhibitory effects on intestinal fibrosis through IL-10. Both M2b and undifferentiated M0 macrophages accumulate among intestinal myofibroblasts. Expression of KLF4 by M0 macrophages that have accumulated in the intestinal myofibroblast environment is suppressed, leading to the release of pro-inflammatory cytokines such as TNF-α and IL-6, in turn causing damage to the intestinal epithelium. The data also suggest that M0 cells accumulating in the intestinal myofibroblast environment also promote the activation of myofibroblasts and intestinal fibrosis by upregulating the expression of IL-36α, a member of the IL-1 family.

## 4. Discussion

In this study, we demonstrated that KLF4 expression differs with each macrophage subtype and revealed that KLF4 expression is high in M0 undifferentiated macrophages as well as M2a and M2c cells. Knockdown of *KLF4* in M0, M2a, and M2c cells induced their conversion into inflammatory macrophages characterized by TNF-α expression. Analysis of the interaction between macrophages and IMFs revealed that M0 and M2b cells exhibit high migration activity toward activated IMF environments, and M2b cells, characterized by IL-10 expression, suppress IMF activation. In an activated IMF environment, *KLF4* and *MRC1* expression were decreased, and *TNF-α* and *IL-6* expression increased in M0 cells, resulting in a transition to an M1-like inflammatory macrophage phenotype. Additionally, IL-36α, a member of the IL-1 family that promotes IMF activation, was upregulated in M0 cells in an activated IMF environment. Knockdown of *KLF4* in M0 cells significantly increased *IL-36α* expression, suggesting that KLF4 regulates the expression of *IL-36α* associated with intestinal fibrosis induction, along with macrophage polarization. Furthermore, using HIOs, we showed for the first time that IL-36γ is produced by cells in an injured human small intestinal epithelium stimulated by TNF-α, leading to IMF activation along with IL-36α release from macrophages, inducing fibrosis.

Previous studies suggested that KLF4 is a critical regulator of macrophage polarization between the M1 and M2a phenotypes. However, the role of KLF4 in other M2 subtypes has not been investigated. In this study, we revealed differences in KLF4 expression in macrophage polarization, including M2b and M2c cells, and demonstrated that *KLF4* knockdown induces the expression of proinflammatory factors such as *TNF-α* in M0 cells as precursors of polarized macrophages and M2c cells. Previous studies showed that the overexpression of *KLF4* suppresses the expression of proinflammatory genes by sequestering co-activators essential for NF-κB activation. Conversely, macrophages deficient in KLF4 exhibit M1-like polarization with expression of inflammation-related genes and bactericidal activity. Thus, KLF4 acts as a switch that controls the phenotype of inflammatory macrophages during macrophage polarization [17,21,22].

In the classical classification scheme, M1 macrophages are characterized by pro-inflammatory effects, whereas M2 macrophages exhibit anti-inflammatory effects and promote wound healing and fibrosis. In this study, we found that M2b macrophages significantly inhibited IMF proliferation, and the migratory capacity of M2b cells was significantly increased under activated IMF conditions. By contrast, M2c macrophages significantly promoted IMF proliferation, whereas the migratory capacity of M2c cells under IMF conditions was unchanged. The results of our study suggest that each M2 macrophage subtype exerts distinct functions during the development of fibrosis in the small intestine. We also demonstrated that IL-10 is highly expressed by M2b cells and inhibits the expression of *αSMA* (Figure 1B and Figure 3F). Therefore, the anti-fibrotic effect of M2b could be mediated by the activation of IL-10 expression. Although some studies have proposed that IL-10 primarily suppresses fibrosis by modulating inflammatory processes that are believed to promote fibrotic proliferation, the precise molecular mechanisms underlying this effect have not been fully elucidated [23,24]. In heart disease, the administration of IL-10 was shown to result in a significant suppression of proinflammatory cytokine synthesis, MMP-9 activity, and the infiltration of inflammatory cells in the myocardium, leading to a reduction in cardiac fibrosis [25,26]. Given that CD is strongly associated with full-thickness damage of the small intestine, resulting in fibrosis and restructuring, targeting IL-10 could be a potential therapeutic approach to prevent the development of fibrosis in intestinal tissue in IBD.

This study focused on the interaction between M0 cells, which are undifferentiated macrophages, and KLF4 in an activated IMF environment. M0 cells showed high migration toward an activated IMF environment along with M2b cells, and the interaction with activated IMFs induced the expression of inflammatory cytokines such as TNF-α via a decrease in *KLF4* expression in M0 cells. These results suggest that undifferentiated macrophages migrating to stromal myofibroblasts surrounding the intestinal epithelium during inflammation might undergo a phenotypic transformation into M1-like macrophages. Furthermore, we found that the interaction with IMFs led to an increase in *IL-36α* expression in M1-like macrophages differentiated from M0 cells. As IL-36α upregulates *αSMA* expression in IMFs, these findings suggest that M1-like macrophages differentiated from M0 cells also play a role in the fibrotic response of the intestine. Indeed, infliximab, an anti-TNF-α antibody, has shown remarkable efficacy in regulating inflammation in IBD patients; however, worsened stenosis of the intestinal tract can occur even after the induction of remission, which may involve IL-36α. *KLF4* knockdown in M0 macrophages significantly increased *IL-36α* expression, suggesting that KLF4 may directly or indirectly regulate *IL-36α* expression. Further studies are needed to elucidate the detailed mechanism regarding how KLF4 regulates IL-36α expression during the polarization of M0 cells to M1-like cells.

IL-36α is a member of the IL-1 family, which includes seven agonists: IL-1α, IL-1β, IL-18, IL-33, IL-36α, IL-36β, and IL-36γ [27,28]. Previous studies showed that in the intestine, IL-36α and IL-36γ expression is upregulated in IBD patients, and experiments using a murine dextran sodium sulfate colitis model showed that the IL-36R signaling pathway plays an important role modulating intestinal fibrosis in chronic inflammation [11,27,29,30,31,32]. This study used human small intestinal organoids derived from human iPS cells to demonstrate for the first time that IL-36γ is upregulated in inflamed epithelium. Given that IL-36γ also increases *αSMA* expression in intestinal myofibroblasts, these data suggest that IL-36γ derived from the intestinal epithelium, as well as IL-36α derived from macrophages, plays a crucial role in the development of fibrosis in the small intestinal epithelium in IBD, including CD. These findings reveal the mechanism by which the interaction between the cytokine network, including members of the IL-1 family, KLF4-induced polarization of macrophages, and activation of stromal myofibroblasts induce intestinal fibrosis in an inflamed small intestinal epithelium in IBD (Figure 5D). The human gut microbiome plays a crucial role in maintaining intestinal homeostasis and regulating immune responses [33]. IBD patients exhibit reductions in microbial biodiversity, leading to imbalances that compromise intestinal integrity and immune function. Additionally, dietary factors, particularly the consumption of ultra-processed foods, have been linked to an increased risk of IBD [34]. Ultra-processed foods, such as soft drinks, refined sweets, salty snacks, and processed meats, have been associated with higher hazard ratios for IBD. Our experimental model could contribute to further research into understanding the complex relationship between environmental factors, diet, and gut microbiota in the development of IBD.

IBD is a chronic and progressive inflammatory disease resulting from an inappropriate immune response [35]. Intestinal fibrosis is a common complication of IBD and typically arises as a result of mesenchymal cell responses to chronic inflammation [36]. Fibrosis is necessary for organ regeneration, including the intestine, as it represents a reparative response to intestinal inflammation, involving tissue remodeling to preserve organ integrity and function. In animal models of colonic damage, spontaneous healing occurs after colonic damage, which contrasts with the persistent inflammation characteristic of clinical IBD [37]. Similarly, in the pancreas, during pancreatic regeneration after repeated episodes of acute pancreatitis and capsaicin administration, temporary atrophy of acinar cells, fibrosis, and the presence of tubular complexes resembling chronic pancreatitis-like lesions were observed [38]. Nevertheless, these lesions regress upon regeneration, suggesting a potential role of fibroblast activity modulation in lesion healing. Understanding these mechanisms may contribute to elucidating why, under normal conditions, the activity of fibroblasts is inhibited after regeneration, leading to the disappearance of morphological symptoms of fibrosis. Thus, elucidating the regulation of fibroblast activity during the regeneration period becomes an important research goal, which could potentially lead to novel therapeutic approaches for fibrosis management in IBD.

There are some limitations of this study. First, THP-1 cells may not fully reflect the differentiation process of normal human macrophages since they originate from acute monocyte leukemia. Due to their leukemia origin, THP-1 cells might exhibit characteristics that differ from those of macrophages differentiating from normal monocytes. In addition, it is important to recognize that their characteristics may not fully represent those of primary human macrophages or other macrophage cell lines. Therefore, the generalizability of our findings to other cell lines or patient populations may be limited. Second, our cultured HIOs possessed the ability to differentiate into enterocytes, goblet cells, enteroendocrine cells, Paneth cells, and mesenchymal cells, which expressed αSMA and vimentin. The upregulation of αSMA in cultured HIOs in response to TNF-α stimulation suggests potential contributions from both the activation of myofibroblasts and the influence of epithelial–mesenchymal transition. Further analyses are required to elucidate the detailed mechanisms underlying intestinal fibrosis. Third, while our study sheds light on the role of KLF4-mediated macrophage polarization in intestinal fibrosis using experimental models, the direct translation of these findings to clinical practice may be limited. The lack of direct investigation in clinical IBD patients and the absence of validation in animal models of IBD indicate the necessity for further studies to confirm the relevance of our observations in the clinical setting.

## 5. Conclusions

In conclusion, our study provided new insights into the role of KLF4 in macrophage polarization and revealed differences in KLF4 expression in M0, M1, M2a, M2b, and M2c macrophage subtypes. Our findings suggest that KLF4 acts as a switch that controls the phenotype of inflammatory macrophages in macrophage polarization. Additionally, we demonstrated that M2b macrophages exert anti-fibrotic effects mediated by the activation of IL-10 expression, whereas M2c macrophages promote IMF proliferation. Moreover, we found that undifferentiated macrophages (M0) can undergo a phenotypic transformation into M1-like macrophages upon interaction with activated IMFs, which also play a role in the fibrotic response of the intestine via regulation of IL-1 family member expression. These results could contribute to the development of potential therapeutic approaches to prevent fibrosis in IBD.

## Data Availability

The data presented in this study are available on request from the corresponding author (Takahito Katano).

## Supplementary Materials

The following supporting information can be downloaded at: https://www.mdpi.com/article/10.3390/medicina60050713/s1, Table S1: Scratched area healing rate of scratch-wound healing assay.

## Abbreviations

CD, Crohn’s disease; Ctrl, control; DAPI, 4′,6-diamidino-2-phenylindole; ECM, extracellular matrix; EGF, epidermal growth factor; FBS, fetal bovine serum; FGF, fibroblastic growth factor; GAPDH, glyceraldehyde-3-phosphate dehydrogenase; HIOs, human-induced pluripotent stem cell-derived small intestinal organoids; IBD, inflammatory bowel disease; IFN, interferon; IL, interleukin; IMF, intestinal myofibroblast; iPS cell; induced pluripotent stem cell; KLF, Krüppel-like factor; LPS, lipopolysaccharide; PBS, phosphate-buffered saline; RPMI, Roswell Park Memorial Institute; RT-PCR, real-time reverse transcription PCR; SD, standard deviation; siRNA, small interfering RNA; TGF, transforming growth factor
